# Kibble-Zurek exponent and chiral transition of the period-4 phase of Rydberg chains

**DOI:** 10.1038/s41467-020-20641-y

**Published:** 2021-01-18

**Authors:** Natalia Chepiga, Frédéric Mila

**Affiliations:** 1 Institute for Theoretical Physics, University of Amsterdam, Science Park 904, Postbus 94485, 1090 GL Amsterdam, The Netherlands; 2 Department of Quantum Nanoscience, Kavli Institute of Nanoscience, Delft University of Technology, Lorentzweg 1, 2628 CJ Delft, The Netherlands; 3 Institute of Physics, École Polytechnique Fédérale de Lausanne (EPFL), CH-1015 Lausanne, Switzerland

**Keywords:** Ultracold gases, Phase transitions and critical phenomena, Theoretical physics

## Abstract

Chains of Rydberg atoms have emerged as an amazing playground to study quantum physics in 1D. Playing with inter-atomic distances and laser detuning, one can in particular explore the commensurate-incommensurate transition out of density waves through the Kibble-Zurek mechanism, and the possible presence of a chiral transition with dynamical exponent *z* > 1. Here, we address this problem theoretically with effective blockade models where the short-distance repulsions are replaced by a constraint of no double occupancy. For the period-4 phase, we show that there is an Ashkin-Teller transition point with exponent *ν* = 0.78 surrounded by a direct chiral transition with a dynamical exponent *z* = 1.11 and a Kibble-Zurek exponent *μ* = 0.41. For Rydberg atoms with a van der Waals potential, we suggest that the experimental value *μ* = 0.25 is due to a chiral transition with *z* ≃ 1.9 and *ν* ≃ 0.47 surrounding an Ashkin-Teller transition close to the 4-state Potts universality.

## Abstract (web summary)

Phase transition occurring in quantum material is an intriguing phenomenon. Here, the authors discuss the commensurate-incommensurate phase transition out of the period-4 phase on a chain of Rydberg atoms and emphasize the emergence of a chiral transition.

## Introduction

Understanding the nature of quantum phase transitions in low-dimensional systems is one of the central topics in condensed matter physics^1,2^. Over the last decades, the combination of conformal field theory in 1+1D^3,4^ and advanced numerical techniques such as the density matrix renormalization group (DMRG) algorithm^5–8^ has proven to be extremely powerful in coming up with theoretical predictions for numerous fascinating critical phenomenas. In that respect, modern quantum simulators based on Rydberg atoms trapped with optical tweezers offer a remarkably rich experimental playground to further investigate quantum physics in 1D. In particular, in a recent experiment^9^, the quantum critical dynamics of a chain of Rubidium atoms ^87^Rb with programmable interactions has been probed. The atoms are excited to a Rydberg state by a homogeneously applied laser with Rabi frequency Ω, while the laser detuning Δ controls the population of excited atoms. The quantum many-body Hamiltonian of the system can be written in terms of hard-core bosons (i.e. bosons with no more than one particle per site) as

1 $${H}_{\text{Ryd}}=\sum _{i}\left[-\frac{{\rm{{{\Omega }}}}}{2}({d}_{i}^{\dagger }+{d}_{i})-{{\Delta }}{n}_{i}+\mathop{\sum }\limits_{R=1}^{+\infty }{V}_{R}{n}_{i}{n}_{i+R}\right]$$

where the van der Waals potential between Rydberg states decays as

2 $${V}_{R}=V{\left(\frac{1}{R}\right)}^{6}.$$

The competition between the detuning term Δ that favors a high density of Rydberg states and the blockade leads to a sequence of lobes of density-wave phases with fixed periodicities. In general, these periodicities can be any rational number, and in the classical limit Ω → 0 they form a Devil’s staircase^10^, but at finite values of Ω, the phase diagram is dominated by lobes of integer periodicities *p* = 2, 3, 4, …^9,11,12^, surrounded at least partially by a critical floating phase for *p* ≥ 3 (ref. ^11^). However, according to recent experiments in which the detuning frequency has been swept for various interatomic distances *a*, this floating phase cannot be present along the whole boundary for *p* = 3 and 4 since a direct transition with a non-integer dynamical exponent *z* larger than 1 has been detected in the vicinity of the tip of the lobe^9^.

The transition out of a period-*p* phase is an example of commensurate–incommensurate transition, a problem with a long history that goes back to the investigation of adsorbed monolayers on surfaces^13–15^. In these systems the role of Rydberg atoms is played by domain walls between periodic phases, and quite remarkably the melting of these periodic phases is a very subtle problem that has not yet received a full solution. For *p* = 2, the transition is known to be generically Ising, while for *p* ≥ 5 it is a two-step process through a Luttinger liquid phase (called a floating phase in the context of adsorbed monolayers) if it is not first order. The difficult cases are precisely *p* = 3 and *p* = 4. In these cases, along the commensurate line, i.e. the line along which, in the disordered phase, the wave vector keeps the value *q* = 2*π*/*p* of the ordered phase, the transition is expected to be continuous in the three-state Potts universality class for *p* = 3, and in the Ashkin–Teller universality class (see below) for *p* = 4. Away from this line, the disordered phase is incommensurate. As pointed out by Ostlund^16^ and Huse^17^, this introduces a chiral perturbation, and the open problem is to understand the effect of this chiral perturbation on the transition.

For *p* = 3, the chiral perturbation is always relevant, and the question is whether it immediately opens a floating phase away from the Potts point, or whether the transition remains direct and continuous for a while, but in a new chiral universality class, as suggested by Huse and Fisher^18^, with a dynamical exponent *z* > 1. Numerical^19–23^ and experimental evidence^14,15^ in favor of this possibility has been obtained in the 1980s and early 1990s in the context of adsorbed layers, and very recently in the context of Rydberg atoms^9,12,24–26^.

For *p* = 4, the situation is even richer because the chiral perturbation is not always relevant^13^. With four degrees of freedom, there is in fact a family of universal classes described by the Ashkin–Teller model in which the local degrees of freedom are described by two Ising spins *σ*_*i*_ ⊗ *τ*_*i*_ coupled by an interaction *σ*_*i*_*σ*_*j*_ + *τ*_*i*_*τ*_*j*_ + *λσ*_*i*_*τ*_*i*_*σ*_*j*_*τ*_*j*_. The asymmetry parameter *λ* controls the relevance of the chiral perturbation. Indeed, according to Schulz^27^, the crossover exponent *ϕ* of the chiral perturbation for the Ashkin–Teller model is given by

3 $$\phi =\frac{3\nu }{2}+\frac{1}{4}-\frac{{\nu }^{2}}{2\nu -1}$$

where *ν* is the exponent of the correlation length The chiral perturbation is relevant if *ϕ* > 0, i.e. if $\nu \, > \, {\nu }_{c}=(1+\sqrt{3})/4\simeq0.683$, irrelevant otherwise. Now, the exponent *ν* is known exactly as a function of *λ*^28,29^:

4 $$\nu =\frac{1}{2-\frac{\pi }{2}{[\arccos (-\lambda )]}^{-1}}$$

At *λ* = 0, the model is known as the four-state clock model and corresponds to two decoupled transverse-field Ising chains. In that case, *ν* = 1: The chiral perturbation is relevant, and it is known to drive the system immediately into a critical phase. By contrast, at the four-state Potts model (*λ* = 1), *ν* = 2/3 < *ν*_*c*_: The chiral perturbation is irrelevant. The critical value of *λ* below which the chiral perturbation becomes relevant is given by

5 $${\lambda }_{c}=-\cos \frac{\pi (\sqrt{3}+1)}{4(\sqrt{3}-1)}\simeq 0.9779$$

**Fig. 1:**
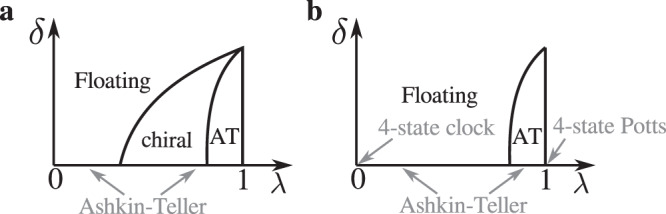
Scenarios for the phase transition in the presence of a chiral perturbation. Sketches of the possible phase diagrams of the transition out of period-4 phase as a function of the parameter *λ* that describes the Ashkin–Teller universality class in the absence of a chiral perturbation, and of the amplitude *δ* of the chiral perturbation **a** with and **b** without a chiral transition. The width of the Ashkin–Teller (AT) phase has been exaggerated for visibility. Along the horizontal axis, the transition is always in the Ahskin–Teller universality class, ranging from the four-state clock model at *λ* = 0 to the four-state Potts model at *λ* = 1.

As long as the chiral perturbation is irrelevant, a line of continuous transition in the Ashkin–Teller universality class can be expected around the commensurate line until the chiral perturbation becomes relevant. Then the situation is similar to the *p* = 3 case, with again the possibility of a chiral transition before a floating phase appears, as emphasized by Huse and Fisher^30^. These two possibilities are summarized in the generic phase diagrams of Fig. 1. In this figure, *λ* denotes the parameter of the Ashkin–Teller model that describes the transition along the commensurate line, and *δ* stands for the amplitude of the chiral perturbation. For Rydberg atoms, *δ* should be understood as the distance to the Ashkin–Teller point along the transition into the disordered phase. For the Ashkin–Teller model itself, the chiral perturbation is given by *δ*(*σ*_*i*_*τ*_*j*_ − *τ*_*i*_*σ*_*j*_), the form used in ref. ^27^ to derive the crossover exponent *ϕ*.

Now, for *p* = 4, the experimental results on Rydberg atoms^9,12^ are compatible with a continuous transition, with a Kibble–Zurek exponent *μ* ≃ 0.25. This exponent is related to *ν* by the relation *μ* = *ν*/(1 + *νz*), where *z* is the dynamical exponent. Since between the clock model (*λ* = 0) and the Potts model (*λ* = 1) the exponent *ν* decreases from 1 to 2/3, the Kibble–Zurek exponent should be between 1/2 and 2/5 if the dynamical exponent was equal to 1. So, according to these experiments, the dynamical exponent has to be larger than 1. This implies that the transition should be a chiral Huse–Fisher transition, i.e., that the scenario of Fig. 1a is realized.

In this paper, we investigate this problem in the context of an effective model for the period-4 phase, the quantum hard-boson model with two-site blockade (see below). We show that: (i) the transition along the commensurate line is sufficiently far from the four-state Potts point to ensure that the chiral perturbation is relevant; (ii) there is an intermediate floating phase far enough from this point; (iii) there is evidence in favor of a small region of chiral transition in between for which we estimate the dynamical exponent and the Kibble–Zurek exponent. Implications for the original model of Eq. (1) and for the experiments on Rydberg atoms are also discussed.

## Results

### The blockade models

Because of the very steep increase of the van der Waals potential at short distance, the simultaneous excitation of atoms at a distance smaller than the so-called Rydberg blockade radius ${R}_{\mathrm{b}}\equiv {({V}_{1}/{{\Omega }})}^{1/6}$ is essentially excluded, a phenomenon known as Rydberg blockade. This means that, on a chain with lattice parameter *a*, the interaction between sites *i* and *j* can be considered to be infinite if *i* − *j* ≤ *r*, where *r* is the largest integer satisfying *r* < *R*_b_/*a*. Keeping only the dominant next-to-blockade repulsion leads to the following effective Hamiltonian:

6 $$H=\sum _{i}-\frac{{{\Omega }}}{2}({d}_{i}^{\dagger }+{d}_{i})-{{\Delta }}{n}_{i}+{V}_{r+1}{n}_{i}{n}_{i+r+1},$$

where *d*_*i*_ (${d}_{i}^{\dagger }$) is an annihilation (creation) operator that acts in a constrained Hilbert space:

7 $${n}_{i}({n}_{i}-1)={n}_{i}{n}_{i+1}=...={n}_{i}{n}_{i+r}=0.$$

We will refer to this model as the *r*-site blockade model. When *r* = 1, it reduces to the original hard-boson model introduced by Fendley et al.^31^. Note also that a constrained Hilbert space equivalent to *r* = 2 has been introduced by Huijse et al.^32^ in the context of a supersymmetric model on a zig-zag ladder. Quite generally, the *r*-site blockade model allows one to discuss period *p* = *r* + 1 and *p* = *r* + 2 phases and their surrounding (see Supplementary Note 1). The main advantage of these constrained models is that their Hilbert space grows much more slowly than that of the original model of Eq. (1), and simulations can be performed on systems large enough to keep track of small changes in the incommensurability and to identify the critical behavior at the transition. Interestingly, the *r*-site blockade model can be seen as the limit of Rydberg atoms with infinitely fast decaying interactions. Indeed, if we consider the model of Eq. (1) with the interaction

8 $${V}_{R}={V}_{r+1}{\left(\frac{r+1}{R}\right)}^{m},$$

the *r*-site blockade model corresponds to *m* → + *∞* while the 1/*R*^6^ model of Eqs. (1) and (2) is recovered for *m* = 6.

### Overview of the phase diagram for *p* = 4

**Fig. 2:**
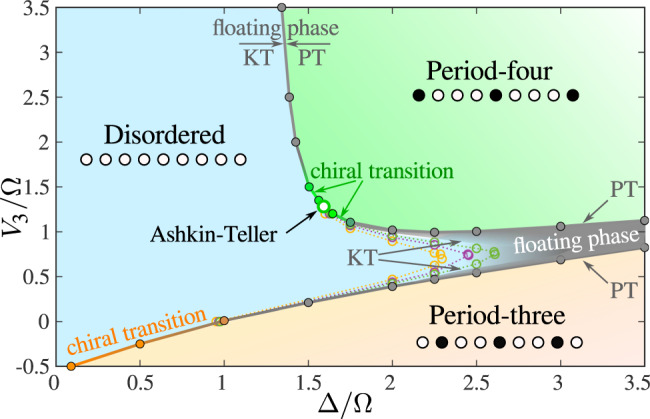
Phase diagram of the hard-boson model with two-site blockade. At the commensurate transition point located at Δ/Ω ≃ 1.593 and *V*_3_/Ω ≃ 1.2839 the transition is in the Ashkin–Teller universality class with *λ* ≃ 0.57 (open green circle). Away from it but not too far (for *V*_3_/Ω ≲ 1.8 and for Δ/Ω ≲ 1.7), our results are consistent with a chiral transition in the Huse–Fisher^30^ universality class. Further away from the Ashkin–Teller point we detect intermediate floating phases bounded by Pokrovsky–Talapov (PT) and Kosterlitz–Thouless (KT) transitions. Above the Ashkin–Teller point the width of the floating phase is always smaller than the width of the gray line. The transition into the *p* = 3 phase is a direct chiral transition from the disordered phase at small Δ/Ω and a PT transition from the floating phase at large Δ/Ω^26^. For large Δ/Ω, the two floating phases adjacent to the *p* = 3 and *p* = 4 phases eventually merge into a single floating phase connecting the two ordered phases. Dotted lines are constant correlation length lines with *ξ* = 50 (yellow), 100 (purple), and 200 (green).

As a first step towards the period-4 phase of Rydberg atoms, let us now turn to the properties of the two-site blockade model. Our numerical results have been obtained with a state-of-the-art DMRG algorithm^5–8^ that explicitly implements the constraints (see “Methods” for details about the algorithm). They are summarized in the phase diagram of Fig. 2. There are three main phases: a disordered phase with incommensurate short-range correlations, and two ordered commensurate phases with period 3 and 4, respectively. Note that these three main phases have been accessed in recent Rydberg atom experiments^9,12^. There are also small floating phases close to the ordered phases. In particular, for large values of Δ, there are two floating phases at the boundaries of the period-three and period-four phases that come closer and create an area of extremely high correlation length. It is therefore probable that the disordered phase eventually disappears and that, for some parameter range, the two ordered phases are connected through a single floating phase, as suggested in refs. ^11,12^ for the model of Eq. (1). Due to the exponential growth of the correlation length at the Kosterlitz–Thouless^33^ phase transition, an accurate investigation of this scenario would require simulations beyond our current limitations.

### Commensurate line

**Fig. 3:**
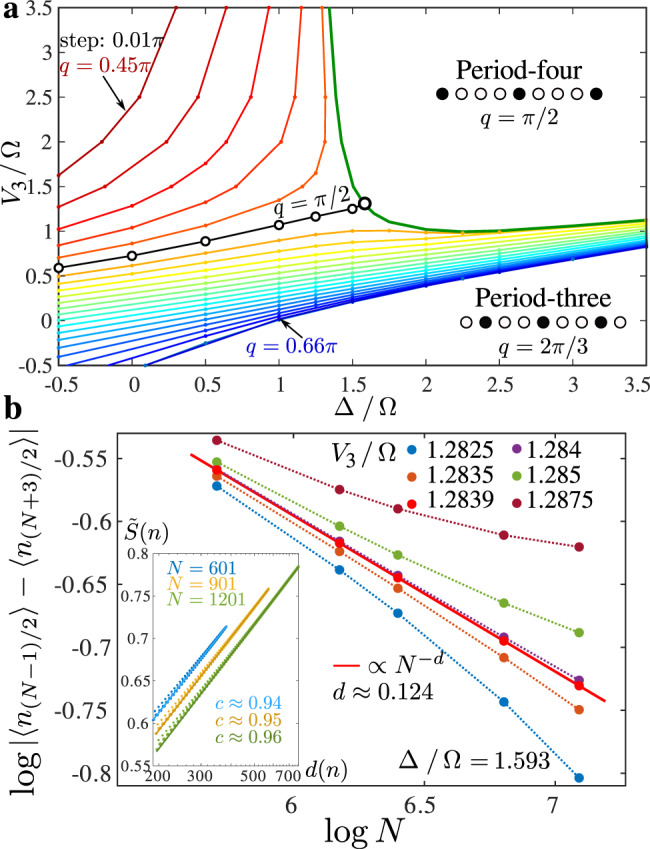
Identification of the conformal point. **a** Phase diagram with equal-*q* lines in the disordered phase extracted for *N* = 601 (systematically) and for *N* = 1201 (in the vicinity of the critical lines). Colors used for equal-*q* lines are guides to the eye. The location of the Ashkin–Teller point has been determined as the crossing point of the critical (green) line and the *q* = *π*/2 line (black, open circles). **b** Finite-size scaling of the amplitude of the oscillations in on-site boson density in the middle of the finite-size chain. The separatrix corresponds to the critical point. Inset: Scaling of the entanglement entropy with the conformal distance *d*(*n*) after removing the Friedel oscillations, leading to a central charge *c* ≃ 0.96.

The transition out of the period-four phase is the main focus of the rest of this section. Our first task is to locate the point on the phase boundary where the chiral perturbation vanishes, hence where the transition can be expected to be described by a conformal field theory. Note that for the original hard-boson model of Fendley et al.^31^ this was not necessary because the three-state Potts belongs to an integrable line, and its location is known exactly. Here this is not the case, but we can expect this point to be located at the intersection of the phase boundary and of the line with wave vector *q* = *π*/2 since along this line the correlations remain commensurate in the disordered phase so that there is no chiral perturbation. To achieve this goal, we have extracted the wave vector *q* (see “Methods”) and have mapped the results over the disordered phase to determine the lines of constant incommensurate wave vector *q*. These lines are depicted in Fig. 3a. The line *q* = *π*/2 enters the period-four phase at Δ/Ω ≃ 1.593. An accurate estimate of the second coordinate has been obtained by a finite-size scaling of the order parameter. It turns out that open boundary conditions favor a boson on the first and last sites. This effectively acts as a conformally invariant fixed boundary condition at the critical point and induces Friedel oscillations in the local boson density. According to boundary conformal field theory, the profile of these oscillations on a finite-size chain is proportional to ${[N\sin (\pi j/N)]}^{-d}$, where the scaling dimension *d* = 1/8 for the Ashkin–Teller model^34^. By scanning *V*_3_/Ω for Δ/Ω = 1.593, we identify a separatrix in the log–log scaling at *V*_3_/Ω = 1.2839 as shown in Fig. 3b. The slope corresponds to *d* ≃ 0.124, in excellent agreement with the scaling dimension *d* = 1/8. As a further check that this is a critical point, we have extracted the central charge by fitting the profile of the reduced entanglement entropy to the Calabrese–Cardy formula (see “Methods”), leading to a central charge *c* ≃ 0.96, within 4% of the conformal field theory prediction *c* = 1.

**Fig. 4:**
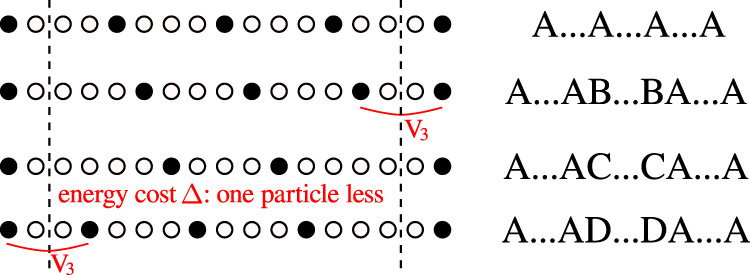
Asymmetry of domain walls in the model with two-site blockade. For *p* = 4, domains with B or D inside A cost an energy *V*_3_ while domains with C inside cost an energy Δ > *V*_3_ since there is one particle less, leading to an asymmetry in the effective transverse field term.

The correlation length of the hard-boson model can be simply obtained by fitting correlations, a straightforward task along the commensurate line (see “Methods”). The resulting correlation diverges at the critical point with an exponent *ν* ≃ 0.78. This is the first indication that *λ* must be significantly smaller than 1. This is actually quite natural. Indeed, when *λ* = 1, the model corresponds to the four-state Potts model with the same amplitude for all flipping processes, while for *λ* < 1 two processes are favored over the third one by the transverse field term. Such an asymmetry naturally appears in the hard-boson model due to the two-site blockade. In the *p* = 4 phase every fourth site is occupied by a boson. So each of the ground states, let us call them A, B, C, and D, corresponds to the location of the occupied sites ${\rm{mod}}\ 4$. From Fig. 4 one can see that domains B and D shifted by one site with respect to the bulk A cost less energy than the domain C shifted by two sites.

One can also estimate *λ* directly by comparing the excitation spectrum of the two-site blockade model with that of the quantum 1D version of the Ashkin–Teller model (see “Methods”). This leads to *λ* ≃ 0.57, in excellent agreement with *ν* = 0.78. At that point, the chiral transition is relevant, with a crossover exponent *ϕ* ≃ 0.33. This means in particular that, away from that point, the transition cannot be a standard continuous transition in the Ashkin–Teller universality class. Either a floating phase opens or the transition becomes chiral.

### Chiral transition versus floating phase

Quite generally, the incommensurate wave vector *q* is expected to approach the commensurate value *π*/2 with a critical exponent called $\bar{\beta }$. To the best of our knowledge the exact value of this critical exponent is not known for the Ashkin–Teller model, but Huse and Fisher^30^ argue that $\bar{\beta } \, > \, \nu$. This implies that the product *ξ* × ∣*π*/2 − *q*∣ decays to zero upon approaching the Ashkin–Teller transition. By contrast, if the transition is chiral, the equality $\bar{\beta }=\nu$ should hold, and *ξ* × ∣*π*/2 − *q*∣ is expected to go to some finite value^30^. When the transition is Ashkin–Teller or chiral, the exponents of the correlation length *ν* in the disordered phase and $\nu ^{\prime}$ in the ordered phase should satisfy $\nu =\nu ^{\prime}$. By contrast, in the presence of an intermediate floating phase, the correlation length in the disordered phase diverges exponentially at a Kosterlitz–Thouless^33^ transition, while the wave vector *q* remains incommensurate, so that the product *ξ* × ∣*π*/2 − *q*∣ diverges. The commensurate–incommensurate transition between the floating and the ordered phases is then expected to be in the Pokrovsky–Talapov^35^ universality class with critical exponent $\bar{\beta }={\nu }^{\prime}=1/2$.

**Fig. 5:**
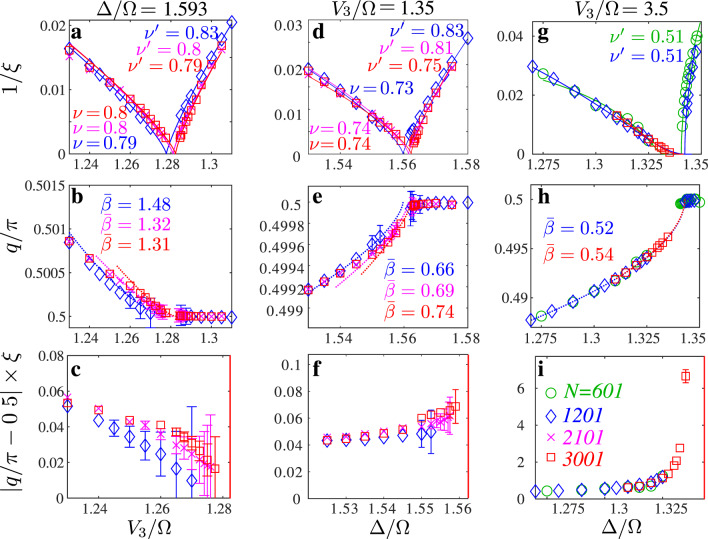
Inverse correlation length 1/*ξ*, wave vector *q*/*π*, and product *ξ* × ∣*π*/2 − *q*∣ along three different cuts across the transition. **a**–**c** Vertical cut through the Ashkin–Teller point at Δ/Ω = 1.593; **d**–**f**, **g–i** Horizontal cuts at *V*_3_/Ω = 1.35 and *V*_3_/Ω = 3.5, respectively. Inside the *p* = 4 phase, the correlation length is fitted with a power law with critical exponent ${\nu }^{\prime}$. In the disordered phase, the correlation length is fitted either with a power law with critical exponent *ν* (**a**, **d**) or with the KT form $\xi \propto \exp ({C}\,/\sqrt{{g}_{KT}-g})$ (**g**), where *g* is the coordinate along the cut. The wave vector *q* is fitted with a power law with exponent $\bar{\beta }$ (dotted lines). Wave vectors *q* are defined within the error bars ±*πξ*/*N*^2^; and *ξ* × ∣*π*/2 − *q*∣ is defined up to ±*πξ*^2^/*N*^2^. For points without error bars, the error bar is smaller than the size of the symbol. In the lower panels, the red lines indicate the boundary of the ordered phase.

In Fig. 5 we take a closer look at three cuts across the transition. Let us start with the vertical cut through the Ashkin–Teller point identified above at Δ/Ω = 1.593. The critical exponents *ν* and ${\nu }^{\prime}$ are in good agreement with each other, and they are also in reasonable agreement with the value obtained for *ν* along the commensurate line and with the value of *λ*. An accurate estimate of $\bar{\beta }$ is very difficult due to the proximity of the commensurate value of *q* in the disordered phase. Nevertheless it is clear qualitatively, just looking at the curvature, that $\bar{\beta }$ is significantly larger than *ν*, in agreement with Huse and Fisher^30^. As a consequence, the product *ξ* × ∣*π*/2 − *q*∣ goes to zero at the critical point as shown in Fig. 5c.

The next cut at *V*_3_/Ω = 1.35, slightly away but very close to this Ashkin–Teller point, is presented in Fig. 5d–f. The correlation length diverges as a power law with similar exponents on both sides of the transition, but, by contrast to the Ashkin–Teller point, the critical exponent $\bar{\beta }$ is much smaller than 1, a clear indication that the chiral perturbation changes the physics immediately away from the Ashkin–Teller point. Its value is comparable to *ν* and ${\nu }^{\prime}$, and accordingly, even if it increases slightly towards the transition, the product *ξ* × ∣*π*/2 − *q*∣ seems to remain finite. The absence of divergence of the product *ξ* × ∣*π*/2 − *q*∣ is a clear indication in favor of the Huse–Fisher universality class. However, as in the case *p* = 3, an extremely narrow floating phase cannot be excluded.

Further away from the commensurate point, the inverse of the correlation length decays in a very asymmetric way, as we show for the horizontal cut at *V*_3_/Ω = 3.5 in Fig. 5g–i. The numerically extracted critical exponent ${\nu }^{\prime}$ is in reasonable agreement with the Pokrovsky–Talapov value 1/2, while the product *ξ* × ∣*π*/2 − *q*∣ clearly diverges towards the transition. By fitting the divergence of the correlation length *ξ* with the predictions for Kosterlitz–Thouless and Pokrovsky–Talapov transitions, we estimate the width of the floating phase to be dΔ/Ω ≈ 3 × 10^−3^. The physics is very similar on the other side of the Ashkin–Teller line (see Supplementary Note 3 for more data).

**Fig. 6:**
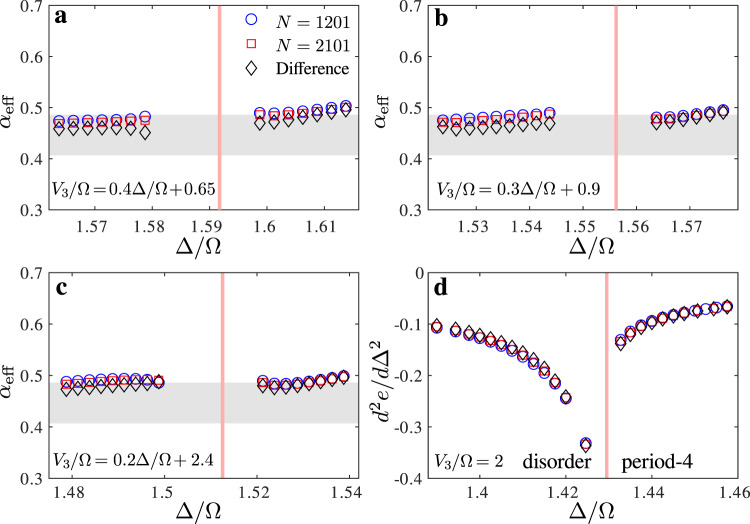
Behavior of the second derivative of the energy close to the transition line. Effective critical exponent *α* across **a** the Ashkin–Teller point and **b**, **c** the chiral transition across oblique cuts perpendicular to the critical line. **d** Second derivative of the energy per site with respect to Δ/Ω for *V*_3_/Ω = 2 around the Pokrovsky–Talapov transition. The results are extracted from the ground-state energy of a chain with *N* = 1201 sites (blue circles) and *N* = 2101 sites (red squares), and from the difference between the two (black diamonds). The gray area indicates the expected value of *α* for a critical exponent *ν* ≈ 0.78 ± 0.02.

As a further check, we have investigated the behavior of the second derivate of the ground-state energy, the equivalent of the specific heat for quantum systems. If the transition is continuous, it is expected to diverge with the same exponent *α* on both sides of the transition, while if there is an intermediate floating phase it is expected to diverge with exponent 1/2 at the Pokrovsky–Talapov transition when coming from the incommensurate phase, and to saturate with a logarithmic singularity on the other side^30^. As can be seen in Fig. 6, the results are fully consistent with a single transition at and close to the commensurate line, and with an asymmetric behavior far enough from it. According to hyperscaling, *α* should be related to *ν* at the Ashkin–Teller point by *α* = 2 (1 − *ν*) ≃ 0.44, in good agreement with the numerical results of Fig. 6a. Interestingly, *α* barely changes as long as the transition is continuous, a fact already noticed and rigorously established for integrable and self-dual versions of the three-state chiral Potts model^36–38^.

### Kibble–Zurek mechanism and dynamical exponent

To estimate the Kibble–Zurek exponent *μ* = *ν*/(1 + *νz*), we need both the dynamical exponent *z* and the correlation length exponent *ν*. Along the commensurate line, the transition is in the Ashkin–Teller universality class and has conformal invariance, so *z* = 1. The estimate *ν* = 0.78 then leads to a Kibble–Zurek exponent *μ* = 0.44. Away from the Ashkin–Teller point, the dynamical exponent *z* can be extracted from *ν* and *α* according to the hyperscaling relation for anisotropic systems: *ν* + *zν* = 2 − *α*. For the cut of Fig. 5d, *ν* = 0.74. Assuming that *α* keeps the value *α* = 0.44 of the Ashkin–Teller point, in agreement with the discussion above, leads to *z* = 1.11. Across this cut, the Kibble–Zurek exponent is thus given by *μ* = 0.41, smaller than across the Ashkin–Teller transition. This conclusion remains true even if we assume that *α* slightly increases away from the Ashkin–Teller point, as suggested by Fig. 6.

## Discussion

To make contact with experiments, let us briefly discuss the implications of the present results for the model with 1/*R*^6^ long-range interactions. As explained above, both models belong to the same family of models defined by Eq. (8). Let us estimate the effect of reducing *m* from +*∞* to 6 for *r* = 2 in the vicinity of the Ashkin–Teller transition. The critical values of the two-site blockade are given by Δ/Ω = 1.593 and *V*_3_/Ω = 1.2839. This value of *V*_3_ corresponds to a Rydberg blockade radius ${R}_{\mathrm{b}}/a=3{({V}_{3}/{{\Omega }})}^{1/6}=3.1276$, near the tip of the *p* = 4 lobe where the experiments have been carried out^9^, and where there is no evidence of a floating phase^11^. The critical value of Δ/Ω = 1.593 is different from that of the Rydberg model at the tip of this lobe (around 2.39), but this is not surprising since Δ is the chemical potential in the bosonic language, and its critical value must be strongly affected by the details of the interactions.

Now let us turn to the nature of the transition, assuming that there is a portion of boundary without floating phase. The physical reason behind *λ* < 1 is the difference in energy cost of domains shifted by one or two sites with respect to the bulk (see Fig. 4). In the very simple classical approximation *δE*_B,D_ ≃ *V*_3_ while *δE*_C_ ≃ Δ. At the Ashkin–Teller critical point, these expressions lead to *δE*_B,D_ ≃ 1.2839 and *δE*_C_ ≃ 1.593 for the blockade model. Taking into account longer-range interactions, the energy of domain walls for the Rydberg model can be estimated as *δE*_B,D_ ≃ *V*_3_ − 2*V*_4_ + *V*_5_ and *δE*_C_ ≃ Δ − 3*V*_4_ + 2*V*_7_. Assuming *V*_3_ = *V* ≃ 1.2839 and a 1/*R*^6^ decay, we get *δE*_B,D_ ≃ 0.885 and *δE*_C_ ≃ 0.925. The asymmetry is still present, but it is smaller, implying that the point where the chiral perturbation vanishes gets closer to the four-state Potts point. Therefore, there are two possibilities: (i) *λ* is still smaller than *λ*_c_ = 0.9779. Then the chiral perturbation remains relevant, and the transition immediately becomes chiral until a floating phase emerges; (ii) the long-range interactions bring the Ashkin–Teller point close enough to the four-state Potts point so that the chiral perturbation is irrelevant; then there will be an extended region of direct Ashkin–Teller transition, followed on both sides by a chiral transition, and ultimately by a floating phase. Since *λ*_c_ ≃ 0.9779 is very close to 1, the first possibility (i) is more likely. More importantly, the fact that the asymmetry can be expected to be reduced by long-range interactions and not increased implies that, if anything, the Rydberg model is further away from the clock limit *λ* = 0 where there would be an intermediate floating phase all along the boundary. So our conclusion that there is a portion of the boundary to the period-4 phase where the transition is direct and continuous in the chiral universality class before a floating phase opens can be considered as a prediction for the Rydberg model with 1/*R*^6^ interactions.

Note that the finite-size effects associated with the restricted number of Rydberg atoms in experiments^9,12^ will, if anything, enlarge the portion without the floating phase. Indeed, if, coming from the disordered phase, the floating phase starts at an incommensurate wave vector *q*, its detection requires the size of the chain to be significantly larger than the period necessary to form at least one helix *N* > 2*π*/(*q* − *π*/2). So, for a finite-size system, the floating phase can only be detected further away from the commensurate line than in the thermodynamic limit, and the transition will look continuous in a larger parameter range, making the observation of this direct transition easier.

Finally, let us discuss briefly the consequences for the Kibble–Zurek experiment. If the transition is chiral, the scaling becomes anisotropic, but if hyperscaling applies, the correlation exponent along the chains *ν* and the dynamical exponent *z* are related by *ν*(1 + *z*) = 2 − *α*. Let us further assume that, as for the two-site blockade model and the self-dual three-state chiral Potts model, the specific heat exponent keeps the value it has at the Ashkin–Teller critical point *α* = 2(1 − *ν*_*λ*_). Then we get *ν*(1 + *z*) = 2*ν*_*λ*_, where the Ashkin–Teller critical exponent *ν*_*λ*_is given by Eq. (4). This implies that *ν* and *z* can be deduced from the Kibble–Zurek exponent *μ* and the asymmetry parameter *λ* according to $\nu =\left[\mu (1+2{\nu }_{\lambda })\right]/(1+\mu )$ and $z=(2{\nu }_{\lambda }-\mu )/\left[\mu (1+2{\nu }_{\lambda })\right]$. Taking the experimental value *μ* ≃ 0.25 and assuming that *λ* is close to 1, as suggested by the small asymmetry of domain walls for Rydberg atoms, we get *z* ≃ 1.9 and *ν* ≃ 0.47. It will be interesting to see if these values can be confirmed by a direct numerical investigation of the model of Eqs. (1) and (2).

## Methods

### Details about the algorithm

**Fig. 7:**
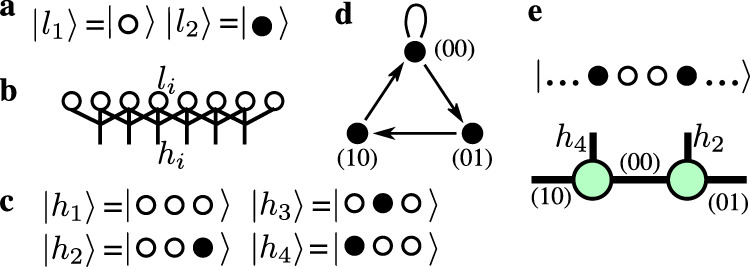
Rigorous mapping onto a model that preserves the block-diagonal structure of tensors. **a** Local Hilbert space of the original model $\left|{l}_{i}\right\rangle$. The open (filled) circle stands for an empty (occupied) site. **b** Rigorous mapping onto a model with a local Hilbert space spanned over three consecutive hard bosons that consist of four states sketched in **c**. The index of the new site corresponds to the index of the middle site. **d** Fusion graph for the recursive construction of the left environment; for the right environment the direction of the arrows should be inverted. **e** Example of the label assignment in MPS representation on two consecutive tensors (green circles) written for the selected state.

The size of the Hilbert space for a model with two-site Rydberg blockade can be calculated using a recursive relation ${\mathcal{H}}(N)={\mathcal{H}}(N-1)+{\mathcal{H}}(N-3)$, with the first three elements of the sequence ${\mathcal{H}}(1)=2$, ${\mathcal{H}}(2)=3$, and ${\mathcal{H}}(3)=4$. So the growth of the Hilbert space with the system size ${\mathcal{H}}(N)\propto 1.46{6}^{N}$ is much slower than ${\mathcal{H}}(N)\propto {2}^{N}$ for an unconstrained model. In order to fully profit from the restricted Hilbert space we implement the blockade explicitly into the DMRG. Recently it has been shown that the hard-boson model with *r* = 1 can be rigorously mapped onto a quantum dimer model on a two-leg ladder^39^ that provides a simple and intuitive way to encode the constraint into DMRG. Although this mapping is not valid for *r* > 1, we can rely on the idea of auxiliary quantum numbers that would preserve the block-diagonal structure of the local tensors. This is achieved by a rigorous mapping onto an effective model that spans the local Hilbert space over three consecutive sites on the original lattice as shown in Fig. 7b. The new local Hilbert space contains four states listed in Fig. 7c. Because of the overlap, the three possible states of two shared sites can be used as a quantum label for the auxiliary bond between two consecutive sites of the new model. By adding a site, for example, by increasing the left environment, one can change the quantum labels according to the fusion graph shown in Fig. 7d. The fusion graph for the right environment can be obtained by inverting the arrows. An example of the label assignment is provided in Fig. 7e.

At the next step, one has to rewrite the hard-boson model given by Eq. (1) of the main text in terms of new local variables $\left|{h}_{i}\right\rangle$. For example, the boson occupation number operator *n*_*i*_, which is also equal to (1 − *n*_*i*−1_)*n*_*i*_(1 − *n*_*i*+1_), can be written in the new local Hilbert space as a 4 × 4 matrix ${\tilde{n}}_{i}$ with the only non-zero element ${\tilde{n}}_{i}(3,3)=1$. The term *V*_3_*n*_*i*−1_*n*_*i*+2_ can be written in the new Hilbert space as a nearest-neighbor interaction ${V}_{3}{\tilde{p}}_{i}{\tilde{q}}_{i+1}$ where the only non-zero matrix elements of the operators $\tilde{p}$ and $\tilde{q}$ are given by $\tilde{p}(4,4)=1$ and $\tilde{q}(2,2)=1$. Finally the constrained flip term

$$-\frac{{{\Omega }}}{2}(1-{n}_{i-2})(1-{n}_{i-1})({d}_{i}^{\dagger }+{d}_{i})(1-{n}_{i+1})(1-{n}_{i+2})$$

can be rewritten as a three-site operator

$$-\frac{{{\Omega }}}{2}({\tilde{a}}_{i-1}{\tilde{b}}_{i}{\tilde{c}}_{i+1}+{\rm{h.c.}}),$$

where the only non-zero matrix elements of the operators $\tilde{a}$, $\tilde{b}$, and $\tilde{c}$ are given by $\tilde{a}(1,2)=1$, $\tilde{b}(1,3)=1$, and $\tilde{c}(1,4)=1$.

With these definitions, the matrix product operator in the bulk takes the following simple form:

9 $$\left(\begin{array}{lllllll}\tilde{I}&.&.&.&.&.&.\\ \tilde{q}&.&.&.&.&.&.\\ \tilde{c}&.&.&.&.&.&.\\ {\tilde{c}}^{\dagger }&.&.&.&.&.&.\\ .&.&\tilde{b}&.&.&.&.\\ .&.&.&{\tilde{b}}^{\dagger }&.&.&.\\ -{{\Delta }}\tilde{n}&{V}_{3}\tilde{p}&.&.&-\frac{{{\Omega }}}{2}\tilde{a}&-\frac{{{\Omega }}}{2}{\tilde{a}}^{\dagger }&\tilde{I}\end{array}\right),$$

where dots mark zero entries of the tensor. Close to the edges one has to carefully modify the MPO to properly encode the boundary terms. This requires the definition of local operators slightly different from those used in the bulk.

There is yet another crucial point that we want to mention. The labels that we have introduced split the Hilbert space into blocks or sectors and therefore correspond to some conserved quantity. For the hard-boson model with a single-site blockade, the quantum labels correspond to the parity of the domain walls. In the present case, the physical meaning of the conserved quantity is not as obvious. However, the only relevant information for us is that the conservation of this abstract quantity requires at least three sites. In other words, by acting with any term (read flip term) on a two-site MPS, one necessary changes one of the out-going labels, while the flip term applied on three consecutive MPS keeps all external labels fixed. As a consequence, neither single- nor two-site DMRG routines are compatible with the presented constraint implementation, and one has to go for at least three-site updates. At a glance this might look costly with a local Hilbert space of dimension 4 since it leads in principle to an MPO operator of size 7 × 7 × 64 × 64. However, taking into account all the constraints on three sites, the projected three-site MPO is only of size 7 × 7 × 9 × 9.

The explicit implementation of two-sites blockade allows us to reach systems with up to *N* = 3001 sites systematically (and *N* = 4801 sites occasionally), keeping up to 2000 states.

### Calabrese–Cardy formula

According to Calabrese and Cardy^40^ the entanglement entropy in finite-size chain with open boundary conditions scales with the block size *l* as

10 $${S}_{L}(l)=\frac{c}{6}{\mathrm{log}}\,d(l)+{s}_{1}+{\mathrm{log}}\,g,$$

where $d(l)=\frac{2L}{\pi }\sin \left(\frac{\pi l}{L}\right)$ is the conformal distance; *s*_1_ and $\mathrm{log}\,g$ are non-universal constants. The presence of Friedel oscillations caused by the fixed boundary conditions is also reflected in the entanglement entropy profile. In order to remove the oscillations we follow ref. ^41^ and construct the reduced entanglement entropy:

11 $${\tilde{S}}_{N}(l)={S}_{N}(l)-\zeta \langle {{\bf{n}}}_{l-1}{{\bf{n}}}_{l+2}\rangle ,$$

where *ζ* is a non-universal constant in front of the leading local correlations between nearest allowed neighbors adjusted to best remove the oscillations. The fits are performed using sites sufficiently far from the edges (*l*, *L* − *l* ≫ 1).

### Comparison with the Ashkin–Teller model and estimate of *λ*

**Fig. 8:**
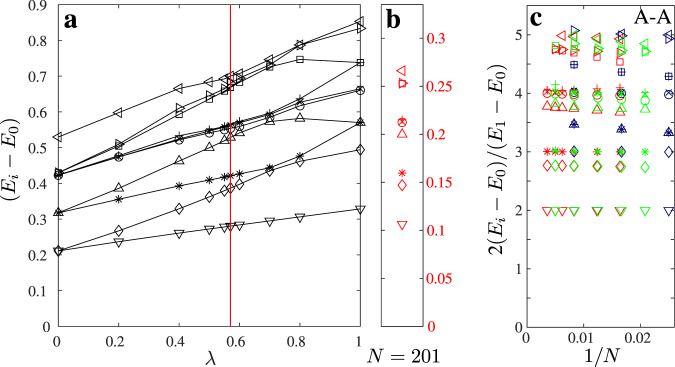
Identification of the Ashkin–Teller parameter *λ* from the conformal tower of states. **a** Excitation spectrum of the Ashkin–Teller model as a function of *λ* for *N* = 60 and A−A boundary conditions (black). Reg line indicate the point where the Ashkin–Teller spectrum resembles the structure presented in **b**. **b** Excitation spectrum of the hard-boson model with *N* = 201 sites (red). **c** Conformal towers of states for hard-boson (red), Ashkin–Teller at *λ* = 0.57 (green), and four-state Potts as Ashkin–Teller with *λ* = 1 (blue). The tower is plotted with respect to the lowest excitation energy.

To estimate *λ* directly, one can compare the excitation spectrum of the two-site blockade model with that of the quantum 1D version of the Ashkin–Teller model defined in terms of Pauli matrices *σ*^*x*,*z*^ and *τ*^*x*,*z*^ by the Hamiltonian:

12 $$\begin{array}{l}{H}_{AT}=-\mathop{\sum }\limits_{j=1}^{N}\left({\sigma }_{j}^{x}+{\tau }_{j}^{x}+\lambda {\sigma }_{j}^{x}{\tau }_{j}^{x}\right)\\ -\beta \mathop{\sum }\limits_{j=1}^{N-1}\left({\sigma }_{j}^{z}{\sigma }_{j+1}^{z}+{\tau }_{j}^{z}{\tau }_{j+1}^{z}+\lambda {\sigma }_{j}^{z}{\tau }_{j}^{z}{\sigma }_{j+1}^{z}{\tau }_{j+1}^{z}\right),\end{array}$$

at its critical point *β* = 1. The spectra have been obtained by targeting several states (up to 11) at every DMRG iteration^42^. In Fig. 8a we show the energy spectrum of the Ashkin–Teller model for *N* = 60 with fixed A–A boundary conditions. We compare these results with the spectrum of the constrained model with *N* = 201 sites. Since the velocity is a non-universal constant, one cannot compare the absolute values of the gap. However we find that the structure of the spectrum in the hard-boson model corresponds to the structure of the Ashkin–Teller spectrum at *λ* ≃ 0.57 (red line in Fig. 8a). In Fig. 8c we further compare the finite-size scaling for hard-boson (red) and Ashkin–Teller model at *λ* = 0.57 (green) and at *λ* = 1 (four-state Potts, blue) and the agreement with *λ* = 0.57 is quite good.

The comparison can be made even more systematic by re-scaling both spectra with respect to the lowest excitation energy as explained in the Supplementary Note 2.

We compute the energy spectrum in a chain with open and fixed boundary conditions. There are two reasons for that. First, DMRG is well known to be more efficient for open boundary conditions than for periodic ones. Second, the number of conformal towers of states that appears in the spectra of periodic or anti-periodic chains are usually larger than the number of towers selected by fixed boundary conditions. However, we have to establish the correspondence between the different boundary conditions in the hard-boson model and in the original Ashkin–Teller model. In the hard-boson model, the simplest way to fix the boundary is to force the first and the last sites to be occupied. If the total number of sites is 4*k* + 1 the same state is favored at each edge, corresponding to the A–A boundary condition in the Ashkin–Teller model. If the total number of sites is 4*k* or 4*k* + 2, we expect A–B and A–D boundary conditions. They are expected to give the same spectrum (assuming that states B and D have equal weight in the transverse field applied on A, while C has a factor *λ*). Finally, if the total number of sites is 4*k* + 3, we expect to observe the spectrum of the A–C boundary condition. Numerical results for A–C and A–B/A–D boundary conditions are provided in the Supplementary Note 2.

**Fig. 9:**
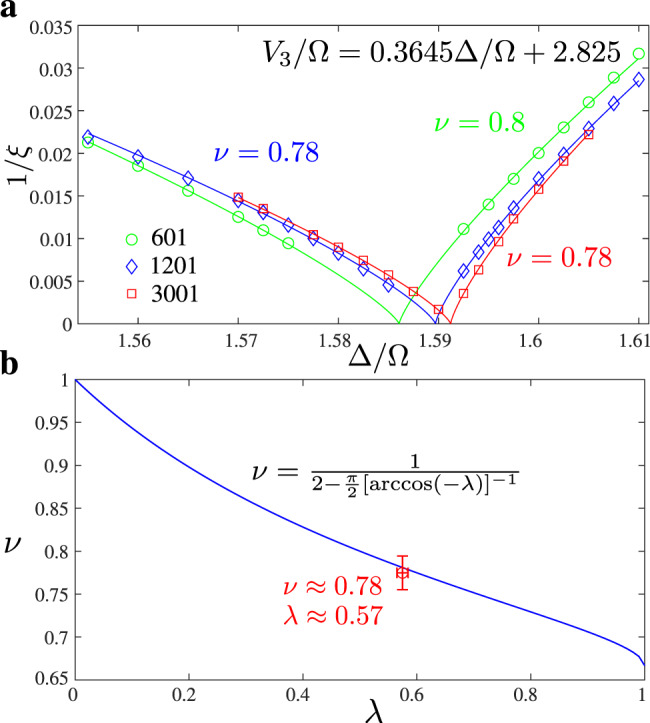
Extraction of the correlation length exponent *ν* and comparison with Ashkin–Teller model. **a** Inverse of the correlation length along the commensurate line. **b** Critical exponent *ν* as a function of the Ashkin–Teller asymmetry parameter *λ*. The dark blue line shows the exact result of refs. ^28,29^. The open red circle is the numerical result of this work *ν* ≃ 0.777 (result for *N* = 3001 shown in panel **a**, finite-size error do not exceed ±0.02) and *λ* ≃ 0.57 ± 0.01 as shown in Fig. 8.

We extract the correlation length critical exponent along the commensurate line which, close to the transition, is given by *V*_3_/Ω = 0.3645Δ/Ω + 2.825. Since we expect a direct transition the critical exponent has to be the same on both sides of the critical point. However, the pre-factor is non-universal. We therefore fit our numerical data with:

$$| x-{{{\Delta }}}_{c}{| }^{\nu }\times [a\theta (x-{{{\Delta }}}_{c})+b\theta ({{{\Delta }}}_{c}-x)],$$

where *a*, *b*, Δ_*c*_, and *ν* are fitting parameters; and *θ*(*x*) is the Heaviside function: *θ*(*x*) = 1 if *x* > 0 and zero otherwise. The results are presented in Fig. 9a.

We compare the values of *λ* and *ν* obtained to fit the hard-boson model with the conformal field theory result of Kohmoto et al.^28,29^ in Fig. 9b. The agreement is very good.

### Extraction of the critical exponent *α*

In order to extract the specific heat critical exponent *α* we look at the divergence of the second derivative of the ground-state energy density *d*^2^*e*/*d*(Δ/Ω)^2^. In order to check the finite-size effects we take the energy per site *e* = *E*/*N* extracted from the total ground-state energy *E* for finite chains with two values of the number of sites: *N* = 1201 and *N* = 2101. We also consider the difference between the two ground-state energies *E*_2101_ − *E*_1201_ to suppress the edge effects and get a better estimate for the bulk energy per site as (*E*_2101_ − *E*_1201_)/900. Approaching the Ashkin–Teller point the specific heat should diverge as ∣Δ − Δ_*c*_∣^*α*^. The effective exponent *α*_eff_ close to the transition can thus be obtained as the slope of $\mathrm{log}\,{d}^{2}e/d{({{\Delta }}/{{\Omega }})}^{2}$ with respect to $\mathrm{log}\,| {{\Delta }}-{{{\Delta }}}_{c}|$. The results are presented in Fig. 6a, where the pink line shows the location of the critical point. According to the hyperscaling relations *α* = 2 − 2*ν* and to our estimate of the correlation critical exponent *ν* ≈ 0.78 ± 0.02, the specific heat critical exponent is expected to be *α* ≈ 0.44 ± 0.04. This corresponds to the gray area in Fig. 6a, showing that our results for *α* are in reasonable agreement with this estimate at the Ashkin–Teller point.

Far enough from the Ashkin–Teller point, the transition is expected to take place through an intermediate floating phase. At the Pokrovsky–Talapov point, the second derivative of the energy is expected to be very asymmetric, with a divergence with exponent 1/2 on the incommensurate side and no divergence on the commensurate side^30^. The results of Fig. 6d obtained for *V*_3_/Ω = 2 are in good agreement with these predictions.

### Extraction of the correlation length and of the wave vector

**Fig. 10:**
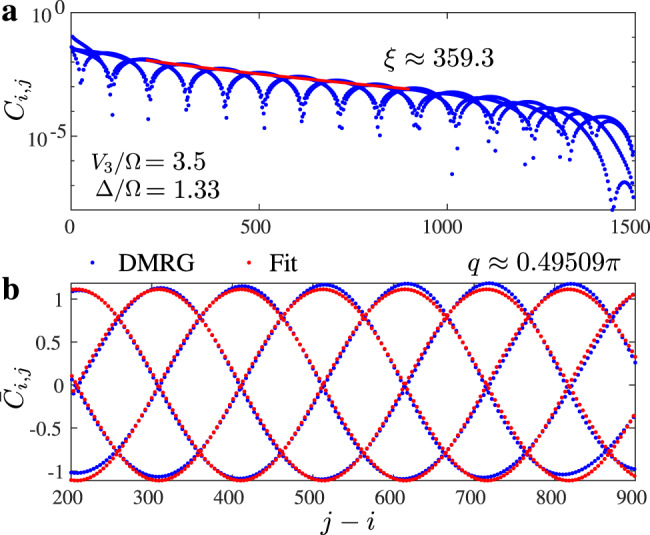
Example of fit of the correlation function to the Ornstein–Zernicke form. In the first step **a**, we extract the correlation length discarding the oscillations. In the second step **b**, we fit the reduced correlation function to extract the wave vector *q*.

In order to extract the correlation length and the wave vector *q*, we fit the boson–boson correlation function to the Ornstein–Zernicke form^43^

13 $${C}_{{\rm{OZ}}}i,j\propto \frac{{e}^{-| i-j| /\xi }}{\sqrt{| i-j| }}\cos (q| i-j| +{\varphi }_{0}),$$

where the correlation length *ξ*, the wave vector *q*, and the initial phase *φ*_0_ are fitting parameters. In order to extract the correlation length and the wave vector with a sufficiently high precision, we fit the correlation function in two steps. First, we discard the oscillations and fit the main slope of the decay as shown in Fig. 10. This allows us to perform a fit in a semi-log scale ${\log}C(x=| i-j| )\approx c-x/\xi -\mathrm{log}\,(x)/2$, which in general provides more accurate estimates of the correlation length on a long scale. Second we define a reduced correlation function

14 $${\tilde{C}}_{i,j}={C}_{i,j}\frac{\sqrt{| i-j| }}{{e}^{-| i-j| /\xi +c}}$$

and fit it with a cosine ${\tilde{C}}_{i,j}\approx a\cos (q| i-j| +{\varphi }_{0})$ as shown in Fig. 10b. The agreement is almost perfect: The DMRG data (blue dots) are almost completely behind the fit (red dots). Fitting the correlations over different windows shows that the error on the correlation length does not exceed 3%, and that the wave vector *q* is determined with a precision O(10^−6^). However, the main source of error in the case of *q* is not the fit itself, but finite-size effects. If the correlation length was infinite, *q* would exhibit finite-size steps of width 2*π*/*N*, leading to an error bar of *π*/*N*. But if the correlation length is smaller than the number of sites, this is a clear overestimate. Indeed the *q* vector adapts close to the boundary, and the steps in the *q* vector in the bulk are significantly rounded and disappear in the limit of small correlation length. To take this effect into account, we include a factor *ξ*/*N* into the error, leading to an error bar of the order *πξ*/*N*^2^.

## Supplementary information

Supplementary Information

## Data Availability

The data that support the findings of this study are available from the corresponding author upon reasonable request.

## Code availability

The code that support the findings of this study is available from the corresponding author upon reasonable request.
