# Supplementary material for: Kibble-Zurek exponent and chiral transition of the period-4 phase of Rydberg chains
Source: Nat Commun. 2021 Jan 18;12:414. doi: 10.1038/s41467-020-20641-y (PMC7814058; doi:10.1038/s41467-020-20641-y)
Supplement: Supplementary file 1 — Supplementary Information [file 41467_2020_20641_MOESM1_ESM.pdf]

Supplementary Material for:
"Kibble-Zurek exponent and chiral transition of the period-4 phase of Rydberg chains"

Natalia Chepiga[1] and Frédéric Mila[2]

[1]*Institute for Theoretical Physics, University of Amsterdam, Science Park 904 Postbus 94485, 1090 GL Amsterdam, The Netherlands*

[2]*Institute of Physics, Ecole Polytechnique Fédérale de Lausanne (EPFL), CH-1015 Lausanne, Switzerland*

SUPPLEMENTARY NOTE 1. BLOCKADE MODEL AND $1/R^6$ POTENTIAL

In this section we further discuss the relevance of the r-site blockade model as an approximation of the Rydberg model with van der Waals interactions. This model is defined by the Hamiltonian in Eqs. (6-7) of the main text.

In the absence of interaction between sites at distance $r+1$ and larger, the model with r-site blockade has two gapped phases - a disordered phase for positive or small negative values of the chemical potential Δ and a period $p = r+1$ crystalline phase that corresponds to the maximally occupied state consistent with the blockade. Upon increasing the repulsion V_{r+1} between sites at distance $r+1$, a period $p = r+2$ phase is expected to emerge. Thus the r-rite blockade model can effectively describe the transition to the $p = r+2$ phase and the region between the $p = r+1$ and $p = r+2$ phases. Note that in the blockade models, and unlike in the Rydberg Hamiltonian of Eqs. (1-2) of the main text, we do not limit V_{r+1} to be strictly positive. In Fig.1 we marked the approximate regions where the blockade model is applicable.

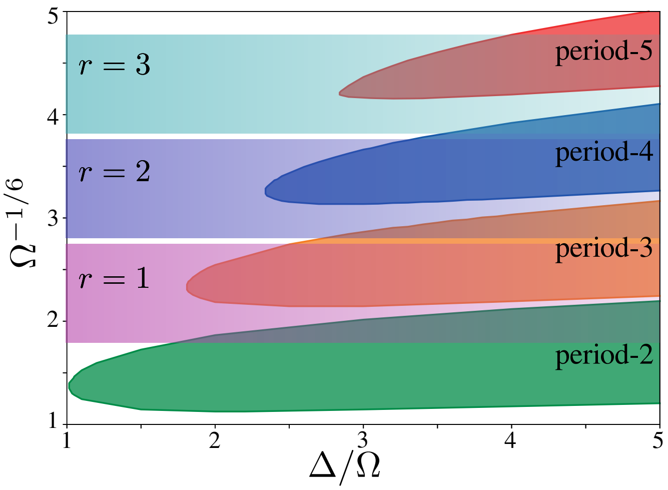

Figure 1. Approximate regions where the blockade model is applicable. The lobes of the ordered phases in the background have been sketched after Refs.1 and 2

.

SUPPLEMENTARY NOTE 2. FURTHER DETAILS ON CONFORMAL TOWERS AT THE ASHKIN-TELLER POINT

Here we show how the conformal towers of the blockade model and of the Ashkin-Teller model can be made systematic. In both the Ashkin-Teller and hard-boson models, the velocity of sound is a non-universal constant. So a quantitative comparison of these two spectra is only possible when the velocity is removed. This we achieve by re-scaling both spectra with respect to the lowest excited state, using a pre-factor 2 for a reason explained below. The crossing point of the two spectra gives the estimate of $\lambda \approx 0.57$ as shown in Fig.2.

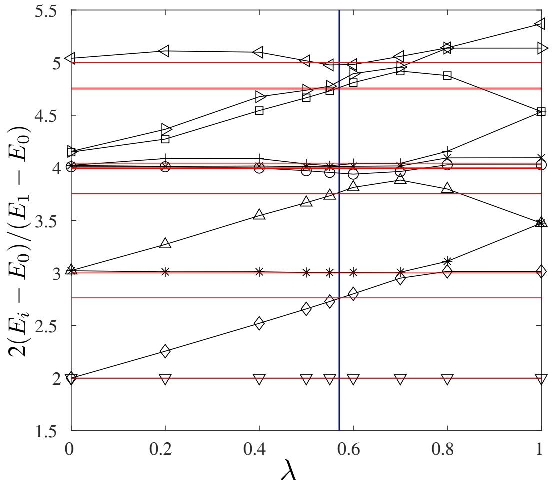

Figure 2. Energy spectrum of the Ashkin-Teller model (black) as a function of λ re-scaled with respect to its lowest state for a chain with $N = 60$ sites and A-A boundary conditions. The results are compared to the re-scaled spectrum of the hard-boson model for $N = 201$ sites with bosons on the first and last sites (red). We extract λ by looking for the value where the spectra match best. Quite remarkably, the crossings of different levels occur for about the same value of λ, supporting the idea that the Ashkin-Teller model is the appropriate model to compare to. The resulting value of λ corresponds to the blue line at $\lambda \approx 0.57$.

We further test the extracted value of λ by looking at the finite-size scaling of the conformal towers of states. The results for A-A boundary conditions have been presented in the main text. In Fig.3 we present the results

for A-B (same as A-D) and A-C boundary conditions. Note that here we do not use the pre-factor 2 for the rescaled spectrum. This is related to the fact that for A-A boundary condition we expect the lowest energy states to be described by the identity conformal tower I for which the $n = 1$ excitation is missing. According to Fig.4(a) of the main text this seems to be the case everywhere for $0 \leq \lambda \leq 1$. To the best of our knowledge the boundary-field correspondence has not been worked out in CFT for the Ashkin-Teller model, so the operator content for A-B, A-C and A-D boundary conditions is not known. However, by analogy with other minimal models, one can expect an equally spaced spectrum for the primary conformal tower, so the lowest state will be $n = 1$.

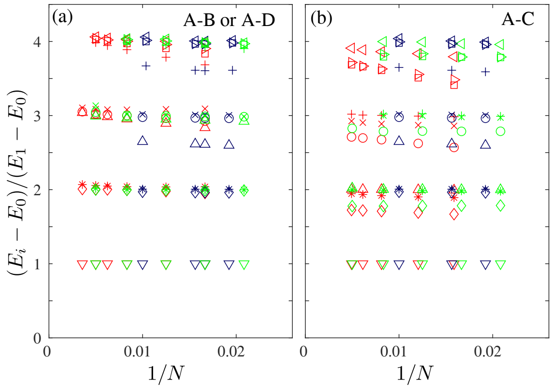

Figure 3. Conformal towers of states at the Ashkin-Teller point for A-B or A-D (a) and for A-C (b) boundary conditions. Red symbols state for the hard-boson data, green for the Ashkin-Teller model at $\lambda = 0.57$, blue for the 4-state Potts model (equivalent to Ashkin-Teller at $\lambda = 1$)

SUPPLEMENTARY NOTE 3. NUMERICAL DATA ALONG A FEW SELECTED CUTS

In the main text we probe the nature of the phase transition to the $p = 4$ phase based on numerical results for the correlation length ξ and the wave-vector q across three selected cuts. The location of these cuts is shown on the phase diagram in Fig.4 by red dashed lines. In Fig.8 we provide data for five more cuts that cross the $p = 4$ phase boundary at different places indicated in Fig.4 by green solid lines.

The data for $\Delta/\Omega = 2$ and for $V_3/\Omega = 2.5$ point towards an intermediate floating phase. The data for $V_3/\Omega = 1.2$ just below the Ashkin-Teller point is consistent with a chiral transition, similar to the data for $V_3/\Omega = 1.35$ just above the Ashkin-Teller point (see main text). The data for $\Delta/\Omega = 1.75$ and $V_3/\Omega = 1.5$ are not conclusive: the product $|q\pi - 1/2| \times \xi$ increases by a factor of ≈ 2 but seems to stay finite. If indeed there is a chiral transition very close to the Ashkin-Teller point,

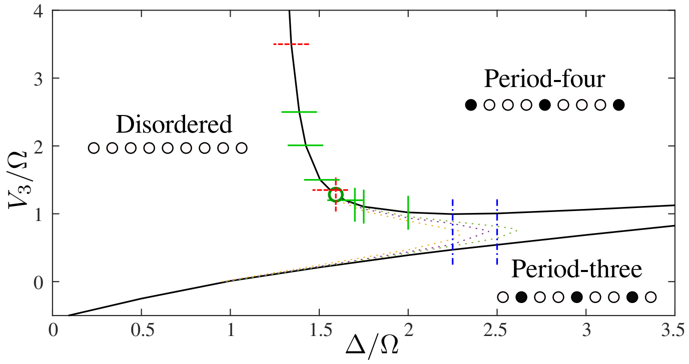

Figure 4. Position of the cuts across the transition to the $p = 4$ phase along which we have investigated the properties of the transition in detail. For clarity the length of each cut is enlarged. Solid black lines indicate the phase boundaries, the open green circle states for the symmetric Ashkin-Teller point. Dotted lines between $p = 3$ and $p = 4$ phases are equal-ξ lines with $\xi = 50$ (yellow), 100 (purple), 200 (green). Red dashed lines mark the cuts considered in the main text. Data across solid green lines are provided in Fig.8. Data along the cuts marked by blue dash-dotted lines are shown in Fig.6.

then these data might signal the proximity to the Lifshitz points at which a floating phase emerges.

In Fig.6 we present the data across two different cuts that connect the $p = 3$ and $p = 4$ phases marked in Fig.4 by blue dash-dotted lines. We see that the correlation length remains very large between the two Pokrovsky-Talapov transitions: for $\Delta/\Omega = 2.5$ it never goes below $\xi = 120$. Beyond $\Delta/\Omega = 2.5$ the finite-ξ region cannot be resolved with our algorithm.

Let us also comment on the transition out of the period-three phase. It is expected to take place either through an intermediate floating phase or through a direct transition in the Huse-Fisher chiral universality class. Since the wave-vector $q > 2\pi/3$ cannot be realized at finite V_3 because of the two-site blockade, the conformal 3-state Potts point is pushed to $V_3 = -\infty$.

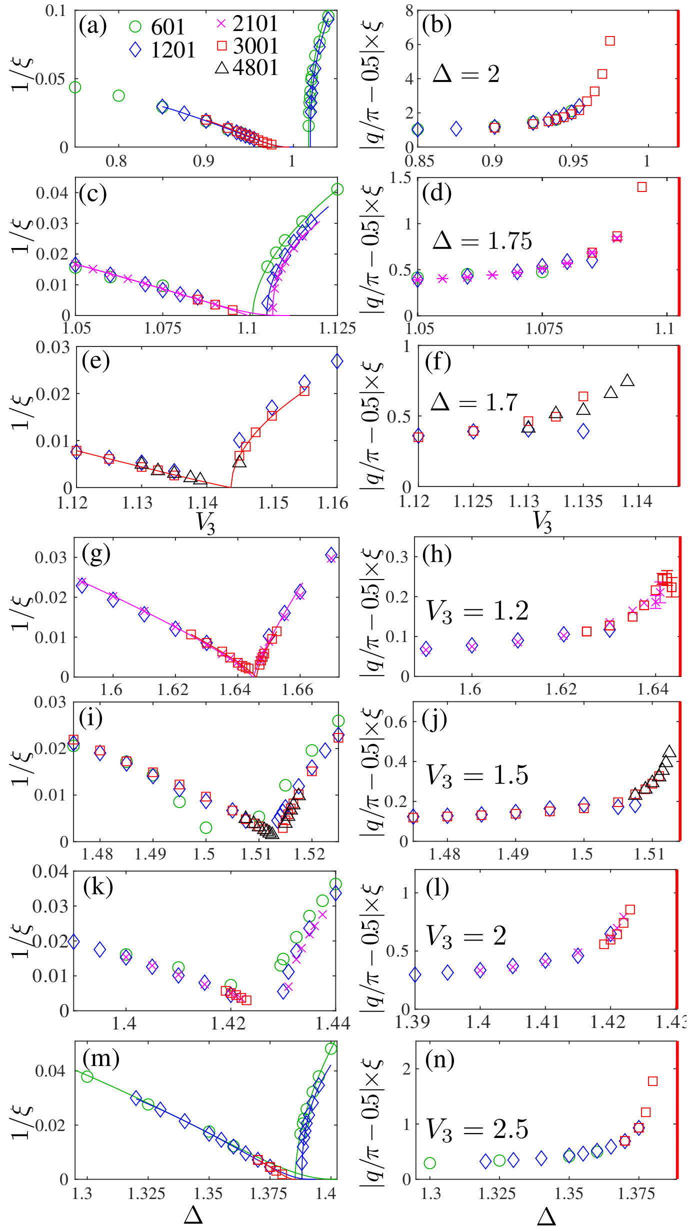

Figure 5. Inverse of the correlation length (left) and product $|q\pi - 1/2| \times \xi$ for a few selected horizontal (a-f) and vertical (g-n) cuts across the $p = 4$ phase transition below (a-h) and above (i-n) the symmetric Ashkin-Teller point. Different symbols correspond to the system sizes listed in (a). The product $\xi \times |\pi/2 - q|$ is defined up to $\pm\pi\xi^2/N^2$. For points without error bars, the error bar is smaller than the size of the symbol.

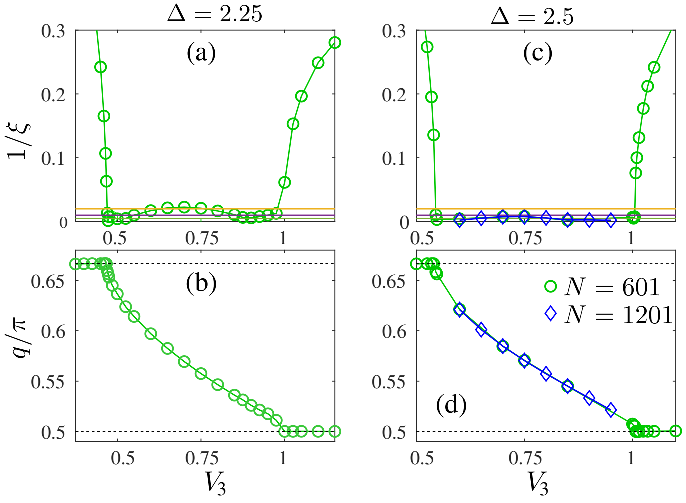

Figure 6. Inverse of the correlation length (left) and wave-vector q (right) across two vertical cuts from the $p = 3$ to the $p = 4$ phase. In (a) and (c) horizontal lines corresponding to $\xi = 50$ (yellow), $\xi = 100$ (purple) and $\xi = 200$ (green) are shown for reference.

SUPPLEMENTARY NOTE 4.

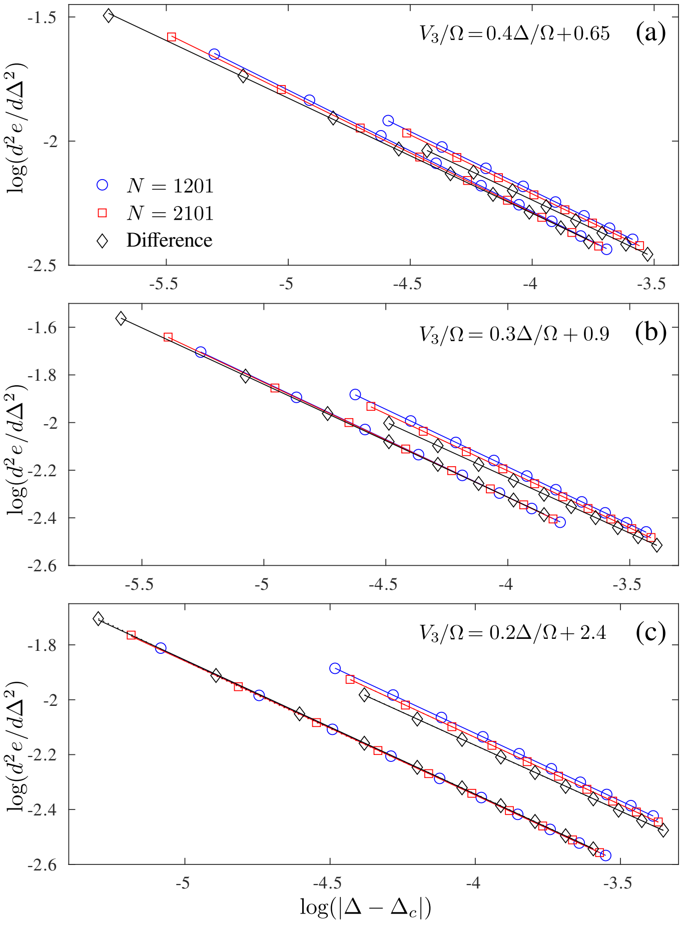

Figure 7. Second derivative of the ground-state energy across the oblique cuts of Fig.6(a)-(c) of the main text on the two sides of (a) the Ashkin-Teller point and (b),(c) the chiral transition.

SUPPLEMENTARY NOTE 5.

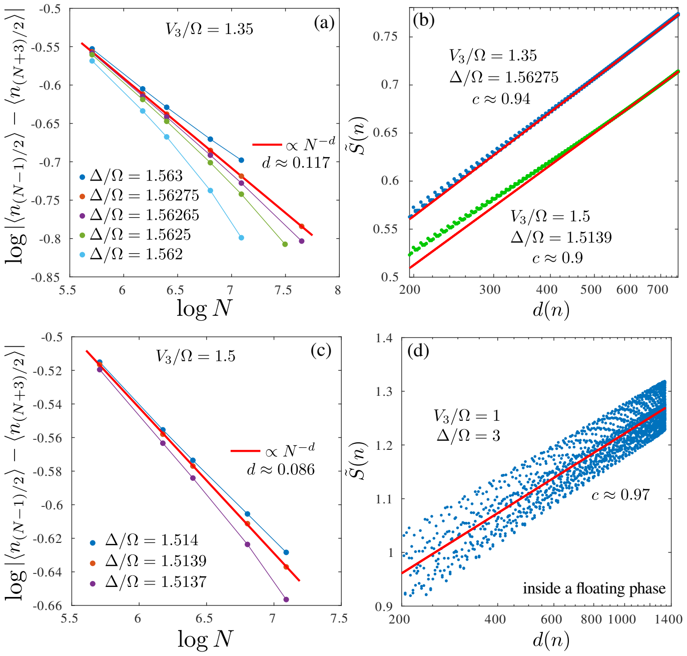

Figure 8. Numerical evidence of that the conformal invariance is lost at the chiral transition. The effective scaling dimension d decays away from the CFT value $d = 1/8$ at the Ashkin-Teller point and takes the value (a) $d \approx 0.117$ at $V_3/\Omega = 1.35$ and (b) $d \approx 0.086$ at $V_3/\Omega = 1.5$. Panel (c) shows the scaling of the reduced entanglement entropy with conformal distance at the critical point at $V_3/\Omega = 1.35$ (blue) and $V_3/\Omega = 1.5$ (green). The effective central charge is also differents from the CFT value $c = 1$ at the Ashkin-Teller point. As shown in panel (d), by contrast, CFT invariance is restored inside the floating phase. Indeed the extracted central charge $c \approx 0.97$ is in good agreement with the value $c = 1$ for a Luttinger liquid.

[1] H. Bernien, S. Schwartz, A. Keesling, H. Levine, A. Omran, H. Pichler, S. Choi, A. S. Zibrov, M. Endres, M. Greiner, V. Vuletic, and M. D. Lukin, Nature **551**, 579 (2017).

[2] M. Rader and A. M. Läuchli, "Floating phases in one-dimensional rydberg ising chains," (2019), arXiv:1908.02068 [cond-mat.quant-gas].
